# Synergistic Enhancement of Phenolic Hydroxyl Content in Lignin via Sequential Hydrothermal and Twin-Screw Extrusion Pretreatment Followed by Aqueous Ethanol Organosolv Extraction

**DOI:** 10.3390/polym18111297

**Published:** 2026-05-25

**Authors:** Fangmin Liang, Ting Jiao, Jian Jiao, Chen Huang, Yan Lv, Yongjun Deng, Qingwen Tian, Ting Wu, Beiping Zhu, Shanming Han, Xuelian Zhou, Hongxiang Zhu, Guigan Fang, Fengshan Zhang, Yanshao Liu, Jingpeng Zhou

**Affiliations:** 1 National Key Laboratory for Development and Utilization of Forest Food Resources, Institute of Chemical Industry of Forest Products, CAF, Key Lab. of Biomass Energy and Material, Co-Innovation Center of Efficient Processing and Utilization of Forest Resource, Nanjing Forestry University, Key Lab. of Chemical Engineering of Forest Products, National Forestry and Grassland Administration, National Engineering Research Center of Low-Carbon Processing and Utilization of Forest Biomass, Nanjing 210042, China; liangfangmin1216@icifp.cn (F.L.);; 2 Shandong Key Laboratory of Biobased Material and Green Pulp Papermaking, Shandong Huatai Paper Co., Ltd., Dongying 257335, China; 3 Shandong Yellow Triangle Biotechnology Industry Research Institute Co., Ltd., Dongying 257335, China; 4 Guangxi Key Lab of Clean Pulp & Papermaking and Pollution Control, College of Light Industry and Food Engineering, Guangxi University, Nanning 530004, China

**Keywords:** lignin extraction, sequential hydrothermal and twin-screw extrusion pretreatment, aqueous ethanol organosolv, phenolic hydroxyl content

## Abstract

Lignin, the second most abundant polymer, remains largely underutilized, with nearly 90% of industrial lignin being combusted for energy due to its low phenolic hydroxyl content and structural heterogeneity of conventional extraction methods. The present study proposes a synergistic extraction method integrating sequential hydrothermal and twin-screw extrusion pretreatment followed by aqueous ethanol organosolv extraction. Three pretreatment strategies—hydrothermal pretreatment, twin-screw extrusion pretreatment, and sequential hydrothermal and twin-screw extrusion pretreatment—were compared. The sequential pretreatment exhibited the most favorable performance. Upon organosolv extraction, a lignin extraction rate of 67.9% was achieved, representing a 20.8% increase over that of the raw material. Extensive *β*-O-4′ bond cleavage during the integrated process liberated phenolic hydroxyl groups. This elevated the total phenolic hydroxyl content to 3.43 mmol·g^−1^, representing a 63.3% increase relative to lignin derived from raw material. Concurrently, this bond cleavage yielded lignin with a narrower molecular weight distribution, indicating enhanced structural homogeneity. Additionally, cellulose retention after lignin extraction reached 88.9%. Mass-balance calculations indicated that 1000 g of raw material yielded 82.5 g of xylose and xylooligosaccharides, 148.2 g of highly active lignin, and cellulose-enriched solid residues, thereby facilitating the comprehensive utilization of the three primary components of lignocellulosic biomass.

## 1. Introduction

As fossil reserves deplete and environmental pressures intensify, global interest in developing renewable, eco-friendly biomass resources has grown [1]. Lignin is the second most abundant natural polymer on Earth. It constitutes approximately 15–30% of lignocellulosic biomass and is essential for the structural integrity of the cell wall. Its structure comprises phenylpropanoid monomers—p-hydroxyphenyl (H), guaiacyl (G), and syringyl (S) units—interconnected via ether and carbon–carbon bonds [2]. The presence of aromatic groups in lignin makes it suitable for the production of resins, carbon products, antioxidants, and pharmaceutical precursors [3,4,5]. Industrial lignin is predominantly obtained as a byproduct of chemical pulping and biorefinery operations, with extraction methods yielding kraft lignin, lignosulfonates, organosolv lignin, enzymatic hydrolysis lignin, and others [6,7,8]. Alkaline extraction is the most widely used approach due to its cost-effectiveness and wide range of applications. It is estimated that over 85% of the approximately 100 million tonnes (dry weight) of lignin produced on an annual basis is derived from the kraft process [9,10]. However, alkaline treatment promotes structural recondensation and reduces reactive functional group content, thereby diminishing lignin reactivity. Consequently, despite its abundance, nearly 90% of available lignin is combusted for energy, with the remainder used in low-value applications such as dispersants and adhesives [5,11,12,13]. The complex structure and low reactivity of lignin thus constrain its high-value utilization. Phenolic hydroxyl groups have been identified as critical determinants of lignin reactivity. They directly influence chemical modification, polymerization, and the development of functional materials [14,15]. Located on aromatic rings, these hydroxyl groups exhibit relative nucleophilicity. They can undergo esterification, etherification, and aldol-type condensations. Consequently, increasing lignin’s phenolic hydroxyl content enhances its chemical activity. This broadens applications in high-value products, including phenolic resins, bioactive materials, antioxidants, and nanomaterial precursors [16,17]. However, current extraction methods seldom attain complete cleavage of ether linkages, resulting in low phenolic hydroxyl content. Moreover, conventional acidic processes tend to promote condensation reactions that diminish lignin homogeneity and restrict its applicability in advanced materials [18]. Complete cleavage of the *β*-O-4 ether bond yields more phenolic hydroxyl groups. The employment of mild organic solvents, such as aqueous ethanol or γ-butyrolactone mixtures, has been demonstrated to be an effective measure in preventing native hydroxyl groups [19].

This study addresses the challenge of lignin utilization, which is constrained by the scarcity of active hydroxyl sites. A combined strategy integrating sequential hydrothermal and twin-screw extrusion pretreatment followed by aqueous ethanol organosolv extraction was proposed to dissociate fibers and increase the content of active phenolic hydroxyl groups of lignin. Initially, mild hydrothermal treatment cleaved some *β*-O-4′ linkages, increasing the phenolic hydroxyl content and partially decomposing large lignin molecules. Subsequently, an organosolv extraction further cleaved *β*-O-4′ bonds and enhanced phenolic hydroxyl content. The effects of the pretreatment processes on fibre composition, lignin structure and yield were investigated. The mechanism by which this synergistic extraction method improved lignin yield and phenolic hydroxyl content was clarified. A comparison of the lignin structure before and after dissociation revealed the mechanisms of dissociation during pretreatment. To validate the synergistic effect of the sequential hydrothermal and twin-screw extrusion pretreatment on lignin extraction rate and structural characteristics, four samples comprising raw material (C), hydrothermally pretreated material (P), twin-screw extruded material (E), and sequentially pretreated material (PE) were compared. Lignin extraction rate, phenolic hydroxyl content, molecular weight distribution, and *β*-O-4′ linkage composition were analyzed following aqueous ethanol organosolv extraction to assess the individual contributions and combined benefits of the pretreatments.

## 2. Materials and Methods

### 2.1. Raw Materials and Reagents

Poplar wood chips (approximately 2.0 × 3.0 × 0.1 cm) were obtained from Shandong Huatai Paper Co., Ltd. (Dongying, China). 2-Chloro-4,4,5,5,-tetramethyl-1,3,2-dioxaphospholane (phosphitylating reagent, TDMP), deuterated dimethyl sulfoxide (DMSO-d_6_), deuterated chloroform (CDCl_3_), cyclohexanol, chromium(III) acetylacetonate, and chromatographic-grade tetrahydrofuran (THF) were purchased from Sigma-Aldrich Co., Ltd. (St. Louis, MO, USA). All other solvents and reagents, including ethanol, acetic acid, pyridine, sulfuric acid, benzene, hydrochloric acid, dioxane, and acetic anhydride, were acquired from Sinopharm Chemical Reagent Co., Ltd. (Shanghai, China) and used without further purification unless otherwise specified.

### 2.2. Pretreatment

#### 2.2.1. Hydrothermal Pretreatment

Hydrothermal pretreatment was conducted in a rotating digester (ZQS1, Northwest Institute of Light Industry Machinery Factory, Xianyang, China). Poplar chips were immersed in water at a solid-to-liquid ratio of 1:5 (*w*/*v*) and processed at 170 °C for 90 min. Upon completion of the reaction, the pressure was released via the relief valve, and the mixture was discharged. Subsequently, solid and liquid fractions were separated using a 300-mesh filter bag. The liquid fraction was retained for subsequent analysis. The solid residue was thoroughly washed with deionized water (prepared using a GREEN-Q3-10T ultrapure water system, Nanjing Yipu Yida Technology Development Co., Ltd., Nanjing, China) and stored at 4 °C for future use. The material that resulted from the hydrothermal pretreatment was designated P.

The hydrothermal pretreatment conditions were selected based on preliminary optimization and literature precedents. It has been established that temperatures below 160 °C result in insufficient hemicellulose removal, while temperatures above 180 °C promote excessive lignin condensation and cellulose degradation. The 90 min residence time was optimized to achieve 80% xylan removal, while minimizing cellulose loss to below 25%. The 1:5 solid–liquid ratio has been demonstrated to ensure adequate heat and mass transfer while maintaining process economy [20].

#### 2.2.2. Twin-Screw Extrusion Pretreatment

**Figure 1:**
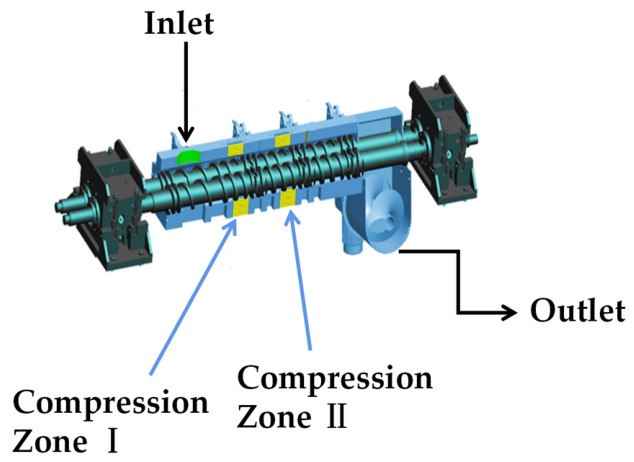
Schematic diagram of the twin-screw extruder.

The poplar wood chips were washed with deionized water and soaked overnight in a 50 L plastic container. After steaming for 15 min, the chips were fed into a twin-screw extruder (Jiangsu Jinwo Machinery Co., Ltd., Jurong, China) at a consistent rate (Figure 1). The extruder was configured with reverse thread clearances of 24 mm in Zone I and 20 mm in Zone II. These settings effectively disrupted fiber bundles and increased specific surface area without causing excessive cellulose fibrillation or energy consumption. The extruded material was collected at the discharge port, with a moisture content of approximately 50%. The material that resulted from the twin-screw extrusion pretreatment was stored at 4 °C for future use and was designated E.

#### 2.2.3. Sequential Hydrothermal and Twin-Screw Extrusion Pretreatment

Poplar wood chips were first subjected to hydrothermal pretreatment as described in Section 2.2.1. After pressure release and filtration, the solid residue was immediately fed into the twin-screw extruder configured with the parameters detailed in Section 2.2.2. The solid material was gathered and stored at 4 °C for subsequent use. The material that resulted from the sequential hydrothermal and twin-screw extrusion pretreatment was designated PE.

### 2.3. Organosolv Lignin Extraction

Lignin extraction was performed in a 1 L high-pressure reactor (YZPR, Shanghai Yan Zheng Instrument Co., Ltd., Shanghai, China) using a 6:4 (*v*/*v*) ethanol–water solvent. The measured masses of four feedstocks were loaded into the reactor: raw material (C), twin-screw extruded material (E), hydrothermally pretreated material (P), and sequentially pretreated material (PE). The solid-to-liquid ratio was 1:10 (*w*/*v*), the reaction temperature was 200 °C, and the reaction time was 60 min. Upon completion, the reactor was rapidly cooled in cold water until the internal pressure equilibrated to atmospheric levels. The reaction mixture was filtered through a Büchner funnel to separate solids from liquids. The solid residual was subsequently rinsed with a fresh solution composed of a 6:4 (*v*/*v*) ratio of ethanol and water. The filtrate and washings were amalgamated and subsequently concentrated to approximately 300 mL, and then diluted with five volumes of deionized water to precipitate lignin. After standing, the supernatant was discarded, and the precipitate was collected by centrifugation. The lignin was washed sequentially with deionized water and freeze-dried (SCIENTZ-10N/C, Ningbo Scientz Biotechnology Co., Ltd., Ningbo, China). The resulting extracted lignins were designated CL, PL, EL, and PEL respectively. The solid residues were further washed with deionized water using a Büchner funnel, then stored at 4 °C for future use, and designated C-EtOH, P-EtOH, E-EtOH, and PE-EtOH, respectively.

### 2.4. Determination of Chemical Components

Materials (50 g each) of raw material (C), pretreated materials (P, E, PE), and solid residues after organosolv lignin extraction (C-EtOH, P-EtOH, E-EtOH, PE-EtOH) were dried at 60 °C in an oven. The dried materials were subsequently ground in a universal high-speed grinder and sieved to obtain particles in the 20–80 mesh range for chemical component analysis. The cellulose, hemicellulose, and lignin components of the original and pretreated biomass materials were determined according to the analytical procedure of the National Renewable Energy Laboratory (NREL). Acid-soluble lignin was measured at 205 nm using a UV–Vis spectrophotometer (T6 New Century UV-Vis Spectrophotometer, Beijing Purkinje General Instrument Co., Ltd., Beijing, China). Monomeric sugars were analyzed by HPLC (Agilent Technologies 1260 Infinity, Agilent Technologies, Santa Clara, CA, USA) equipped with a Bio-Rad Aminex HPX-87H column and using 5 mM sulfuric acid as the mobile phase. All analyses were performed in triplicate to ensure precision. The data were presented as mean ± standard deviation (SD). Statistical significance was considered for *p*-values less than 0.05.

The lignin extraction rates were calculated according to Equation (1).

(1) $$\mathrm{Lignin}\;\mathrm{extraction}\;\mathrm{rate}\;(\%)=\frac{C_{in}-Y\times C_{ex}}{C_{in}}\times 100\%$$

where *C_in_* is the lignin content in the original biomass, %; *C_ex_* is the component content in the biomass after lignin extraction, %; *Y* is the solid yield after lignin extraction, %.

### 2.5. Analysis of Physical Properties of Different Pretreated Materials

#### 2.5.1. Brunauer–Emmett–Teller (BET) Analysis

The pore size and specific surface area of the treated materials were determined using an surface area and porosity analyzer (ASAP 2020, Micromeritics Instrument Corp., Norcross, GA, USA). Prior to analysis, the materials underwent a degassing process at 120 °C for 12 h to remove any adsorbed species (such as moisture or volatile substances). Nitrogen adsorption–desorption isotherms were measured at 77 K (liquid nitrogen temperature) to evaluate the pore characteristics of the fibers. The specific surface area was calculated using the Brunauer–Emmett–Teller (BET) equation, and the pore size distribution was determined using the Barrett–Joyner–Halenda (BJH) model applied to the adsorption branch of the isotherm.

#### 2.5.2. Scanning Electron Microscope (SEM)

Raw material (C) and hydrothermally pretreated wood chips (P) were sectioned transversely into thin slices. Representative specimens of twin-screw extruded material (E) and sequentially pretreated material (PE) were also selected for analysis. All materials were freeze-dried, mounted on aluminum stubs using conductive carbon tape, and sputter-coated with gold for 60–80 s at 10–15 mA to achieve a coating thickness of 10–20 nm. The coated materials were subsequently imaged using a scanning electron microscope (SEM, JSM-7600F, JEOL Ltd., Tokyo, Japan) at an accelerating voltage of 5 kV.

### 2.6. Analysis of Lignin Structure

#### 2.6.1. Separation and Purification of Milled Wood Lignin (MWL)

Milled wood lignin was isolated following an optimized purification protocol. Raw wood chips (C) and the other three pretreated materials (P, E, PE) were ground to pass through a 20-mesh screen using a high-speed grinder (HFW80, Hengfeng Coal Quality Analysis Equipment Co., Ltd., Hebi, China). The powder was then extracted with a benzene-alcohol solution (2:1, *v*/*v*) to remove fats, waxes, and other extractives. The extractive-free wood powder (25 g) was milled in a planetary ball mill (QM-QX, Nanda Instrument Factory, Nanjing, China) equipped with a 500 mL zirconia tank and zirconia grinding balls (100 g total, 1:1:1 ratio of large, medium, and small balls) at 500 rpm. To prevent oxidation, milling was performed in cycles of 10 min operation followed by 10 min interruption, for a total duration of 72 h.

The ball-milled materials were extracted using a 96:4 (*v*/*v*) dioxane-water solution at 50 °C for 48 h with a solid concentration 5% concentration. Following each extraction, the mixture was centrifuged at 9000 rpm for 10 min (CenLee2300R, Hunan Xiangli Scientific Instrument Co., Ltd., Changsha, China), and the supernatant was retained. The extraction was repeated three times. The combined extracts were concentrated to approximately 100 mL by rotary evaporation (RV 10 Digital, IKA Werke GmbH & Co. KG, Staufen, Germany) at 35 °C under reduced pressure. The concentrated solution was added dropwise to 95% ethanol (three times the volume of the extract) with continuous stirring to precipitate hemicellulose. After centrifugation, the hemicellulose precipitate was discarded, and the lignin-containing liquid was further concentrated to 100 mL. The lignin solution was then added dropwise to acidic water (pH 2.0, adjusted with hydrochloric acid) at a volume ten times that of the solution under stirring. The precipitated lignin was collected by centrifugation, washed with deionized water, and freeze-dried to yield crude MWL. The crude MWL was further purified according to a conventional MWL purification procedure to obtain the final purified MWL product.

#### 2.6.2. Structural Analysis of Lignin

The molecular weight analysis of lignin was initially performed by acetylating lignin in accordance with established methods in the literature [21]. Lignin (0.5 g) was dissolved in 6 mL of pyridine–acetic anhydride (1:1, *v*/*v*) and stirred at room temperature for 72 h in the dark. The reaction was quenched by the addition of 10 volumes of hydrochloric acid solution (pH < 2), and the acetylated lignin was precipitated. The precipitated lignin was then separated using a 0.22 μm membrane, washed with ultrapure water, freeze-dried, and stored for subsequent analysis.

For GPC analysis, acetylated lignin (2 mg) was dissolved completely in 2 mL of chromatographic-grade tetrahydrofuran (THF, HPLC grade). The resulting solution was filtered through a 0.45 μm organic syringe filter into a chromatographic vial. The injection volume was set at 10 μL, and the chromatographic analysis was conducted using a PLgel Mixed-D (300 × 7.5 mm) column (Agilent, Santa Clara, CA, USA). The number–average molecular weight (*M*n) and weight-average molecular weight (*M*w) were calculated based on the calibration curve of general-purpose polystyrene.

Lignin structure was characterized by two-dimensional heteronuclear single quantum coherence nuclear magnetic resonance (2D-HSQC NMR) using a Bruker AVANCE III HD 600 MHz NMR spectrometer (Bruker Corporation, Billerica, MA, USA). The acquisition parameters included a spectral width of 166 ppm with 256 data points for the F1(^13^C) dimension and a spectral width of 12 ppm with 1024 data points for the F2 (^1^H) dimension. Other parameters comprised a coupling constant of 145 Hz, a pulse delay of 1.0 s, and 128 scans. For sample preparation, dried lignin (150 mg) was transferred to a 5 mL amber glass vial. Deuterated dimethyl sulfoxide (0.6 mL) was added, and the mixture was vortexed until homogeneous. Ultrasonication was applied to ensure complete dissolution. The dissolved material was then transferred to a nuclear magnetic resonance (NMR) tube. Signal assignment in the HSQC spectra was performed according to previous literature [22]. Semi-quantitative analysis of the HSQC signals was performed using TopSpin 3.5 (Bruker Corporation, Billerica, MA, USA), as described elsewhere [23].

The hydroxyl content of lignin was assessed using a Bruker AVANCE III HD 400 MHz NMR spectrometer (Bruker Corporation, Billerica, MA, USA), following established procedures [24]. Sample preparation was performed as follows: (1) Solution 1 was prepared by mixing anhydrous pyridine and deuterated chloroform (1.6:1, *v*/*v*). (2) Solution 1 (25 mL) was mixed with 100 mg of cyclohexanol (as an internal standard) and 90 mg of chromium acetylacetone (as a relaxation reagent) in a brown reagent bottle to form Solution 2. (3) 10 mg of completely dry lignin was accurately weighed and transferred into a 2 mL glass vial. Solution 1 (400 μL) and Solution 2 (150 μL) were then added to the glass vial. (4) The mixture was thoroughly stirred until the lignin was completely dissolved. Complete dissolution was visually confirmed before proceeding. (5) Prior to analysis, 70 µL of phosphitylating reagent (2-chloro-4,4,5,5-tetramethyl-1,3,2-dioxaphospholane, TMDP) was added, and the solution was stirred for approximately 1 min to ensure homogeneous reaction. The sample was transferred to an NMR tube and analyzed within 1 h.

## 3. Results

### 3.1. Effect of the Hydrothermal and Twin-Screw Extrusion Pretreatment on Lignin Extraction Yield

**Figure 2:**
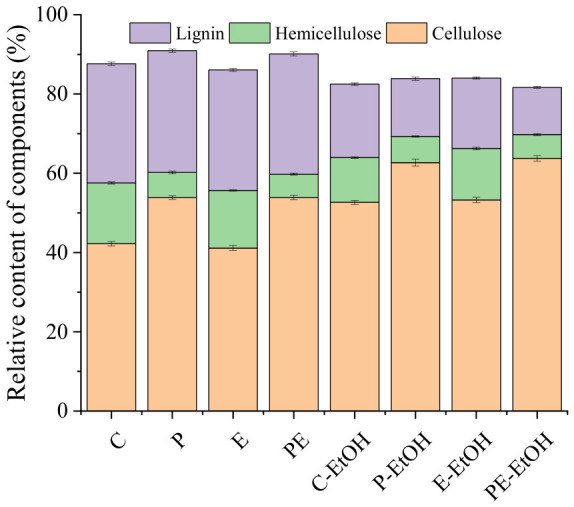
Chemical compositions of materials under different pretreatments and after lignin extraction. Raw material (C); hydrothermally pretreated material (P); twin-screw extruded material (E); sequentially pretreated material (PE); and corresponding solid residues after organosolv lignin extraction (C-EtOH, P-EtOH, E-EtOH, PE-EtOH). Error bars represent standard deviations (*n* ≥ 3).

As shown in Figure 2, the raw material (C) was composed of cellulose (42.2%), hemicellulose (15.4%), and lignin (30.1%). Hydrothermal pretreatment and sequential pretreatment reduced hemicellulose content to 6.4% (P) and 5.9% (PE), respectively, corresponding to removal efficiencies of 58.4% and 61.7%, consistent with previous reports for hydrothermal pretreatment [20]. The concomitant lignin contents increased to 30.7% (P) and 30.3% (PE), reflecting preferential hemicellulose solubilization rather than lignin enrichment. In contrast, twin-screw extrusion pretreatment induced minimal changes in chemical composition. The essential equivalence in chemical composition between PE and P indicates that extrusion functions as a physical refinement step without additional chemical modification. These findings are consistent with previous reports indicating that hydrothermal pretreatment primarily removes hemicellulose while partially degrading and solubilizing lignin.

Comparison of the raw material (C) with the solid residue after organosolv lignin extraction (C-EtOH) revealed that lignin content decreased from 30.1% to 18.6%, and hemicellulose content decreased from 15.4% to 11.3%. The solubilization of hemicellulose during organosolv lignin extraction occurred in accordance with principles analogous to those of hydrothermal pretreatment. High-temperature water self-ionizes to generate H_3_O^+^, promoting deacetylation and release of acetic acid, which further catalyzes glycosidic bond hydrolysis. Furthermore, the use of ethanol reduced the system’s dielectric constant and enhanced the solubility of organic polysaccharides and lignin [25]. Consequently, the cellulose content increased from 42.2% to 52.7%, primarily due to the removal of lignin and hemicellulose, which elevated the relative proportion of cellulose. Compared the hydrothermally pretreated material (P) with the residue solid P-EtOH, hemicellulose content remained stable (6.4% to 6.1%), as labile hemicelluloses were already removed during hydrothermal pretreatment. Lignin content decreased from 30.7% to 14.6%, while cellulose content increased from 53.8% to 62.7%.

**Figure 3:**
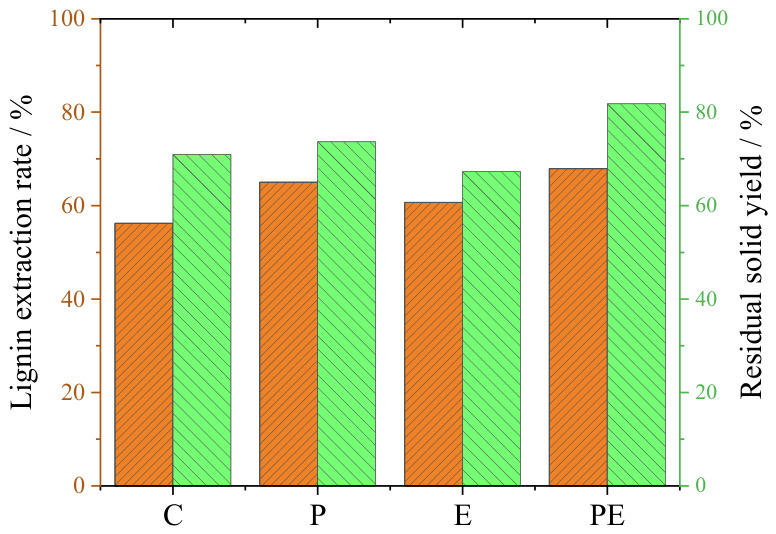
Lignin extraction rate and residual solid yield following organosolv lignin extraction. Raw material (C); hydrothermally pretreated material (P); twin-screw extruded material (E); sequentially pretreated material (PE).

As shown in Figure 3, all pretreatment methods enhanced lignin extraction rates in the organosolv lignin extraction system. The sequential hydrothermal and twin-screw extrusion pretreatment was found to be the most effective, achieving a lignin extraction rate of 67.9%, representing a 20.8% increase over the untreated feedstock (56.2%). Hydrothermal pretreatment and twin-screw extrusion pretreatment yielded extraction rates of 65.0% and 60.7%, respectively. It is noteworthy that the residual solid yield following organosolv lignin extraction was highest for the PE-EtOH (81.8%), followed by P-EtOH (73.7%), C-EtOH (71.0%), and E-EtOH (67.3%). In addition, the cellulose content in the PE-EtOH was found to be highest at 63.9%, indicating that the sequential pretreatment effectively preserves cellulose during the organosolv lignin extraction process, thereby facilitating the integrated utilization of the three major biopolymers.

### 3.2. The Effect of Different Treatments on the Physical Properties of Materials

**Figure 4:**
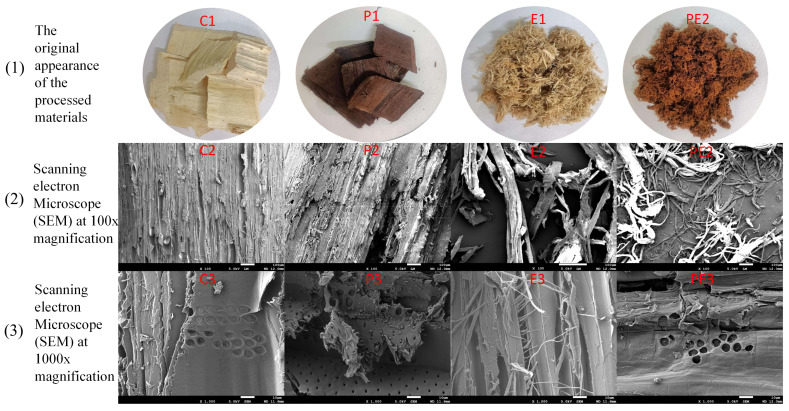
Physical morphology of raw material and pretreated materials: (**1**) Macroscopic appearance; (**2**,**3**) SEM images. Raw material (C); hydrothermally pretreated material (P); twin-screw extruded material (E); sequentially pretreated material (PE).

As shown in Figure 4, the raw material (C) exhibited pale coloration with densely packed fiber bundles and predominantly closed pits under SEM. Hydrothermal pretreatment darkened the chips to brown without altering macroscopic morphology. SEM images revealed retention of dense fiber bundle arrangement, with numerous spherical pseudo-lignin particles deposited on fiber surfaces at 1000× magnification [26,27]. Micropore formation was evident, enhancing liquid penetration and lignin extraction efficiency. Twin-screw extrusion pretreatment transformed the material from chips to large fibrous aggregates. Upon magnification, the compact chips appear to be fragmented into smaller fiber bundles. This phenomenon was attributed to the combined compressive and frictional forces within the twin-screw extruder. The fiber surfaces displayed microfibrillation, which significantly increased the specific surface area, promoted liquid penetration, and consequently enhanced lignin extraction (Figure 3). Following the sequential pretreatment, the chips produced a finer brown fibrillar material in comparison to that produced by twin-screw extrusion pretreatment. SEM indicated further reduction in fiber bundles and development of numerous large surface pores. Previously closed pits opened, substantially increasing specific surface area, porosity, and mass-transfer efficiency. These phenomena were considered to be the primary factors contributing to the observed increase in lignin extraction yield.

**Figure 5:**
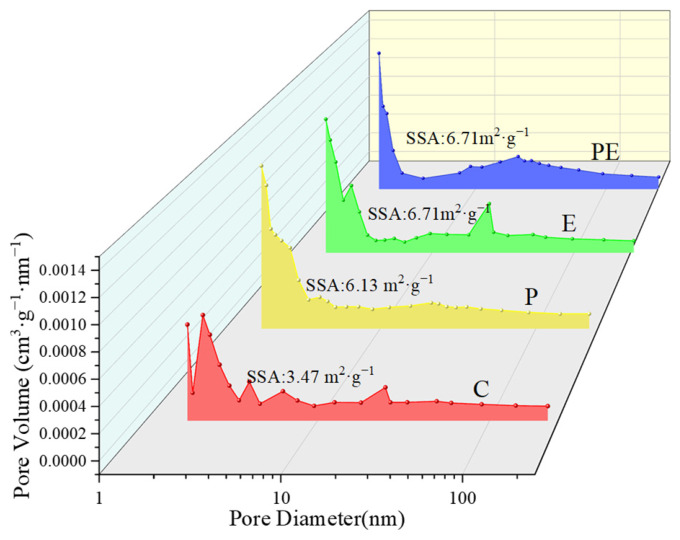
Pore size distribution of raw and pretreated materials. Raw material (C); hydrothermally pretreated material (P); twin-screw extruded material (E); sequentially pretreated material (PE).

To systematically compare the pore-structural characteristics of the four materials, BJH adsorption pore volume distributions were analyzed (Figure 5 and Table 1). All four materials exhibited characteristic microporous to mesoporous structures, with dominant pore sizes concentrated at the 1.8–2.0 nm range. However, notable differences were observed among the materials in terms of pore-size distribution breadth, peak morphology, and the development of hierarchical porosity. These differences reflect the distinct impacts of different pretreatment processes on feedstock pore architecture. Raw material (C) possessed a multimodal pore-size distribution: a principal peak at 2.0–3.0 nm, a secondary distribution at 4.0–6.0 nm, minor shoulders at 20–30 nm, and a weak tail at 50–100 nm. These features originated from cell wall pits, intracellular voids, and interparticle packing gaps. The limited pore volume and small pore size restricted solvent penetration into the secondary cell wall, thereby impeding lignin dissolution.

Compared with raw material C, hydrothermally pretreated material (P) exhibited the most concentrated pore-size distribution: a sharp, highest-intensity main peak (approximately 1.2 × 10^−3^ cm^3^·g^−1^·nm^−1^) appeared at 1.8 nm (micropores), with almost no subsidiary peaks. This indicated a more consistent and uniform pore structure, which was attributed primarily to the removal of hemicellulose during hydrothermal pretreatment. This process disrupted the compact connections among the major cell-wall components and generated numerous fine pores. The specific surface area increased by 76.7% (from 3.47 to 6.13 m^2^·g^−1^). As shown in Figure 5, there was a clear indication of substantial formation of fine pores within the cell walls; such increased porosity was conducive to mass transfer. Extruded material (E) displayed a primary peak at 1.5–2.0 nm with increased peak volume (0.7 × 10^−3^ to 1.1 × 10^−3^ cm^3^·g^−1^·nm^−1^). The rise in micropores was primarily attributed to the collapse of certain pores during extrusion, leading to the formation of smaller pores. A secondary peak of notable intensity was observed at approximately 25.0 nm, while the peak in the 4.0–10.0 nm range essentially vanishes. The peak around 25.0 nm was enhanced (from 0.15 × 10^−3^ to 0.36 × 10^−3^ cm^3^·g^−1^·nm^−1^), primarily due to the cracking or mesopore formation induced by compressive and frictional forces during the extrusion process. As extrusion is a physical process, pore size distribution remained largely unchanged overall; however, increased pore volume at equivalent sizes indicated higher pore density. Fibril disintegration and fibrillation increased specific surface area by 93.4% (from 3.47 to 6.71 m^2^·g^−1^).

Compared with material P, PE exhibited an invariant primary peak at 1.8 nm with comparable micropore volumes, indicating that hydrothermal pretreatment generated similar micropore sizes and quantities prior to extrusion. In contrast, the secondary peak at approximately 20.0 nm displayed a substantially higher volume in PE than in P, demonstrating that extrusion following hydrothermal pretreatment generated additional mesopores. Concurrently, the small peak at approximately 4.0 nm diminished markedly after extrusion, suggesting the transformation of these mesopores into larger mesopores (approximately 20.0 nm) during the extrusion process. Such enlarged mesopores facilitated medium transport. Variations in pore size and number directly affected mass transfer and reaction kinetics, constituting the primary factors contributing to the subsequent differences in lignin extraction yields.

All pretreatment methods enhanced the lignin extraction rate, with the sequential pretreatment exhibiting the most significant effect, achieving a 67.9% lignin extraction rate. During hydrothermal pretreatment, hemicellulose was degraded into oligosaccharides that dissolved in the pretreatment liquor, thereby increasing the porosity of the pretreated biomass and facilitating subsequent lignin extraction. The primary mechanism by which twin-screw extrusion pretreatment enhanced lignin extraction involved the compressive and shear forces within the twin-screw extruder, which partially dissociated fiber bundles and collapsed plug-like pit structures, resulting in the creation of new micropores and an increase in both porosity and specific surface area [28]. The sequential pretreatment surpassed the effects of either hydrothermal pretreatment or twin-screw extrusion pretreatment alone, illustrating a synergistic effect: during the initial hydrothermal process, fibers absorbed water and swelled, which amplified the mechanical action of extrusion. The removal of hemicellulose disrupted the dense cellulose–hemicellulose–lignin network, thereby improving pore structure by increasing the number and volume of pores, expanding pore diameter, and enhancing the material’s specific surface area [29]. The high solid recovery and elevated lignin extraction yield achieved through PE indicated that this pretreatment not only enhanced lignin recovery but also minimized cellulose loss, thereby promoting the comprehensive utilization of the fibrous feedstock.

### 3.3. Enhancement of Phenolic Hydroxyl Content in the Lignin via Sequential Hydrothermal and Twin-Screw Extrusion Pretreatment Followed by Aqueous Ethanol Organosolv Extraction

Milled wood lignin (MWL), produced via mild ball milling and organic solvent treatment during extraction, retains the native lignin structure and functional group characteristics to a significant degree. Consequently, it has been widely accepted as a reference standard in lignin research [30]. Compared with lignin extracted using strong acids, strong bases, or high-temperature methods, MWL undergoes minimal chemical structural disruption and remains closer to its native state. This quality renders it suitable as a benchmark for comparative analysis of the impacts of various extraction or modification methods on lignin structure. Furthermore, MWL demonstrates strong representativeness and reproducibility, which facilitates the establishment of standard lignin structural models and improves comparability across different studies. Thus, it serves as a crucial reference for elucidating lignin structure, assessing reactivity, and advancing new technologies [31]. In this study, milled wood lignin from raw material (C-MWL) and milled wood lignin from P material (P-MWL) were employed as standards. The structural changes and dissociation mechanisms of lignin during hydrothermal pretreatment and aqueous ethanol organosolv extraction were investigated by comparing molecular weight, hydroxyl content, and linkage contents among different MWLs and extracted lignins.

#### 3.3.1. Mechanism of Lignin Dissociation During Hydrothermal Pretreatment

To investigate lignin dissociation during hydrothermal pretreatment, milled wood lignin was isolated from raw material (C-MWL) and hydrothermally pretreated material (P-MWL). As shown in Table 2, the molecular weight increased after hydrothermal pretreatment (8126 to 9266 g·mol^−1^), while the polydispersity index decreased from 5.29 to 3.15. These changes indicated that condensation reactions during hydrothermal pretreatment increased the lignin molecular weight, and that concurrent dissolution and removal of low-molecular-weight fragments narrowed the molecular weight distribution [32].

As shown in Table 3, C-MWL contained more guaiacyl-derived phenolic hydroxyls than syringyl-derived phenolic hydroxyls. This composition reflected cell-wall anatomy: the middle lamella is dominated by G-type units, while the secondary wall is enriched in S-type units [33]; because C-MWL is derived mainly from the middle lamella, its G-type content was relatively high [34]. After hydrothermal pretreatment, the aliphatic hydroxyl content of P-MWL decreased slightly, whereas the total phenolic hydroxyl content increased by 66.4% (1.34 to 2.23 mmol·g^−1^). The primary reason was that the high-temperature aqueous phase facilitated the hydrolysis and cleavage of the *β*-O-4′ bond in lignin under mildly acidic conditions, transforming the aromatic ring end groups previously shielded by ether bonds into liberated phenolic hydroxyl groups. Moreover, following hydrothermal pretreatment, the increase in S-type phenolic hydroxyl groups (0.64 mmol·g^−1^) surpassed that of G-type phenolic hydroxyl groups (0.20 mmol·g^−1^), and the S/G ratio of MWL increased from 1.06 to 1.77. This phenomenon arose primarily because the S-type unit, having been methoxylated at the C5 position, lacks free sites; consequently, it is more prone to ether bond hydrolysis under hydrothermal conditions. In contrast, the G-type unit, which lacks a C5 methoxy group, is more susceptible to condensation reactions. This difference ultimately led to a greater increase in S-type phenolic hydroxyl groups compared to G-type phenolic hydroxyl groups [35,36]. Two-dimensional NMR spectroscopic analysis (Table 4) supported this finding. Following hydrothermal pretreatment, the *β*-O-4′ bond content decreased by 36.30% (from 44.8% to 28.59%) compared to C-MWL, while the C-C bond content increased by 64.19%. This observation further validated the occurrence of condensation reactions within lignin during hydrothermal pretreatment, leading to an increase in the weight-average molecular weight of milled wood lignin. The increase in lignin molecular weight can be further elucidated as follows.

Under typical hydrothermal conditions, hemicellulose degradation generates organic acids, primarily acetic and formic acids, which lower the pH to approximately 3–4 and thereby promote cleavage of lignin *β*-O-4′ linkages and release of phenolic compounds [37]. Based on this analysis, the mechanism for lignin dissociation during hydrothermal pretreatment is proposed as follows. (1) A proportion of water molecules undergo ionization, resulting in the formation of H_3_O^+^ and OH^−^. Simultaneously, acetyl groups (–COCH_3_) on hemicellulose side chains, especially xylan, are cleaved at elevated temperature to release acetic acid (CH_3_COOH), which then ionizes to yield H^+^. Together, these processes produce a mildly acidic medium [38]. (2) The acidic environment catalyzes heterolytic cleavage of lignin ether bonds, generating benzylic carbocation intermediates that are subsequently hydrolyzed, thereby increasing the content of phenolic hydroxyl groups (Table 3) [39]. Depolymerization generates carbocation intermediates that can undergo C–C coupling with electron-rich aromatic rings on neighboring lignin units, producing stable condensed structures [40]. Condensation mainly targets the electron-rich guaiacyl (G) units, which reduces G-unit abundance and thus explains the observed increase in the S/G ratio after hydrothermal pretreatment. During hydrothermal pretreatment, lignin undergoes a dynamic balance of depolymerization, condensation, and redistribution; its structural evolution is jointly driven by chemical bond cleavage, physical migration, and interactions among components [41]. Therefore, controlling pretreatment temperature and residence time to maximize *β*-O-4′ bond cleavage while limiting lignin condensation promotes an increase in lignin phenolic hydroxyl content [42].

#### 3.3.2. Mechanism of Lignin Dissociation in Aqueous Ethanol Organosolv Process

Following different pretreatments, lignin was extracted using an aqueous-ethanol (40:60, *v*/*v*) organosolv system at 200 °C for 60 min with a solid-to-liquid ratio of 1:10. Extraction conditions were optimized through preliminary experiments and literature precedent [43]. The aqueous-ethanol ratio provided an optimal balance between lignin solubility and cellulose preservation. At lower temperatures, the lignin extraction rate was relatively low, whereas excessively high temperatures induced non-productive lignin condensation.

**Figure 6:**
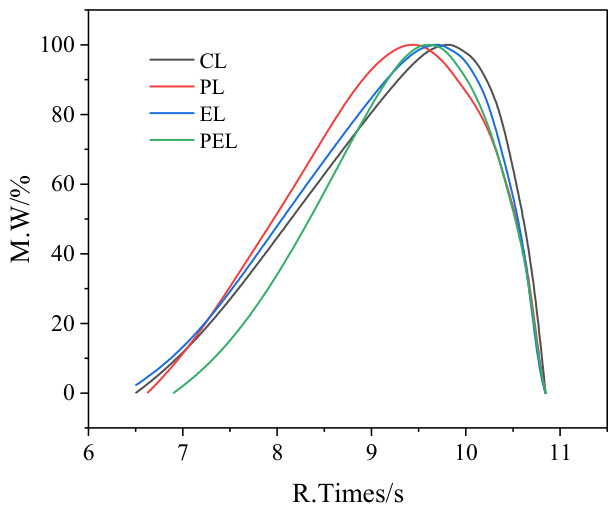
Molecular weight distribution of the extracted lignins (GPC). Lignin extracted from raw material (CL); lignin extracted from hydrothermally pretreated material (PL); lignin extracted from twin-screw extruded material (EL); and lignin extracted from sequentially pretreated material (PEL).

As shown in Figure 6, all four extracted lignins exhibited a characteristic monomodal molecular weight distribution, with overlapping primary molecular weight ranges. This observation suggested that the core components did not undergo significant changes in molecular weight during extraction; however, variations were evident in the proportion of low-molecular-weight fractions and the breadth of the molecular weight distributions. While hydrothermal pretreatment enhanced lignin molecular weight through condensation reactions, PL exhibited a slight reduction in molecular weight compared to CL, along with a decreased polydispersity index (Table 2). PL showed a lower relative weight percentage in the high-molecular-weight region (time < 7.3 s) compared to CL. Conversely, in the moderately high-molecular-weight region (7.3 s < time < 9.7 s), PL exhibited a higher relative weight percentage than CL. Moreover, in the low-molecular-weight region (time > 9.7 s), PL again showed a lower relative weight percentage than CL. These observations indicated that lignin condensation during hydrothermal pretreatment was limited. Compared with CL, EL exhibited a slightly lower molecular weight and a marginally higher polydispersity. These changes were primarily attributed to alterations in the porosity of the fibrous feedstock caused by extrusion, which enhanced solvent penetration and promoted lignin cleavage. Although the extraction process of PEL involved only an additional physical extrusion step compared to PL, it resulted in a 28.86% decrease in weight-average molecular weight. Furthermore, the polydispersity index declined from 2.62 to 2.30, and the molecular weight distribution narrowed (Table 2), displaying the sharpest peak in Figure 6 and demonstrating superior homogeneity, which is beneficial for the integrated utilization of lignin. The enhancement of pore structure and the increase in specific surface area facilitated complete contact between ethanol and lignin in the raw materials, thereby improving the dissociation effect. Additionally, the degraded lignin dissolved quickly in the ethanol solvent, which helped prevent excessive depolymerization of lignin.

The hydroxyl content of extracted lignins was evaluated via P^31^-NMR, as shown in Table 3. The aqueous ethanol organosolv extraction process significantly enhanced the content of phenolic hydroxyl groups. Compared with C-MWL, CL exhibited a 56.72% increase in phenolic hydroxyl content (from 1.34 to 2.10 mmol·g^−1^), while PL exhibited a 39.46% increase (from 2.23 to 3.11 mmol·g^−1^) relative to P-MWL. The increment in S-type phenolic hydroxyls (0.52 mmol·g^−1^ and 0.67 mmol·g^−1^, respectively) surpassed that of G-type phenolic hydroxyls (0.11 mmol·g^−1^ and 0.09 mmol·g^−1^, respectively). The 2D-NMR results indicated that the of *β*-O-4′ bond content decreased by 48.48% and 51.35%, respectively, transforming the aromatic ring end groups masked by ether bonds into free phenolic hydroxyl groups and increasing the phenolic hydroxyl content [40,44]. During hydrothermal pretreatment, partial cleavage of the *β*-O-4′ bond occurred, which reduced further cleavage during organosolv extraction. Consequently, the increase in phenolic hydroxyl content in PL was less pronounced than that in lignin extracted from raw material. The greater increase in S-type phenolic hydroxyl groups compared to G-type phenolic hydroxyl groups was attributed to the structural differences between the two lignin types. The G-type structure was more susceptible to condensation and re-polymerization into insoluble substances than the S-type structure. In contrast, the depolymerization products of S-type units remained stable in the organosolv extraction process. As a result, S-type units were relatively enriched in the solvent, leading to a significant increase in the S/G ratio. Specifically, the S/G ratio of CL increased by 1.65 times compared to C-MWL, whereas the S/G ratio of PL remained essentially unchanged relative to P-MWL. The primary factor influencing this process was the selectivity observed during the hydrothermal pretreatment. During this phase, the structurally unstable and highly reactive fraction of lignin was preferentially degraded and removed, likely resulting in an enrichment of easily cleaved S-type units [45]. Simultaneously, some G-type units may have undergone initial condensation reactions. The difference in reactivity between S-type and G-type units was diminished relative to that of raw material, thereby reducing the “escape” advantage of S-type units and potentially constraining the condensation space available for G-type units [46]. As a result, the ratio of S-type to G-type units in the PL became more similar to P-MWL.

Among all extracted lignins, PEL exhibited the highest phenolic hydroxyl content at 3.43 mmol·g^−1^, representing a 63.3% increase compared to EL. Relative to previously reported methods for lignin extraction from wood, which typically yield phenolic hydroxyl content below 3.1 mmol·g^−1^ and lignin extraction rates of approximately 50% (Table 5) [47,48,49,50,51], PEL demonstrated markedly higher phenolic hydroxyl content and lignin extraction rate. The primary distinction between PEL and EL lies in the inclusion of an additional hydrothermal pretreatment for PEL. This pretreatment promoted cleavage of the *β*-O-4′ bond, thereby contributing to the elevated phenolic hydroxyl content in PEL. Compared to PL, the phenolic hydroxyl group content in PEL increased by 10.29%, with S-type phenolic hydroxyl groups increasing by 10%. Moreover, the concentration of *β*-O-4′ bonds in PEL was marginally higher than that in PL. Notably, PEL underwent an additional physical extrusion process during pretreatment, which primarily involved mechanical shear aimed at enhancing the specific surface area of the fibers and modifying the pore structure. Mesopores predominated, while a relatively high proportion of macropores improved solvent permeability and accessibility. This enhancement facilitated solvent penetration into the secondary walls, which are rich in S-type units, thereby increasing the content of S-type phenolic hydroxyl groups. Furthermore, this process allowed for the effective penetration of hydrated hydrogen ions and solvents into the cell wall interior, while the degraded lignin was promptly dissolved in the solvent, reducing ineffective lignin condensation. Consequently, although the content of *β*-O-4′ bonds in PEL was slightly greater than that in PL, its phenolic hydroxyl content remained higher than that of PL. The combined and synergistic effects of hydrothermal pretreatment and mechanical extrusion collectively enhanced the phenolic hydroxyl content of the extracted lignin.

Studies have demonstrated that aqueous ethanol solvents exhibit distinctive properties at elevated temperatures. At 200 °C, the dielectric constant of a 70% ethanol solution decreases to 30, compared with 55 for pure water [52]. This property enhances the lignin solubility [53]. The oxidation of ethanol yields acetaldehyde and acetic acid, creating a weakly acidic environment (pH ≈ 3.5–4.0) that facilitates the hydrolysis of ether bonds. Furthermore, the hydroxyl oxygen in the ethanol molecule possesses a lone pair of electrons, rendering it an effective nucleophile. Under acid-catalyzed conditions, lignin depolymerization generates electrophilic benzylic carbocation intermediates. Ethanol participates in the reaction through nucleophilic attack, leading to a process known as ethanolysis [54].

**Figure 7:**
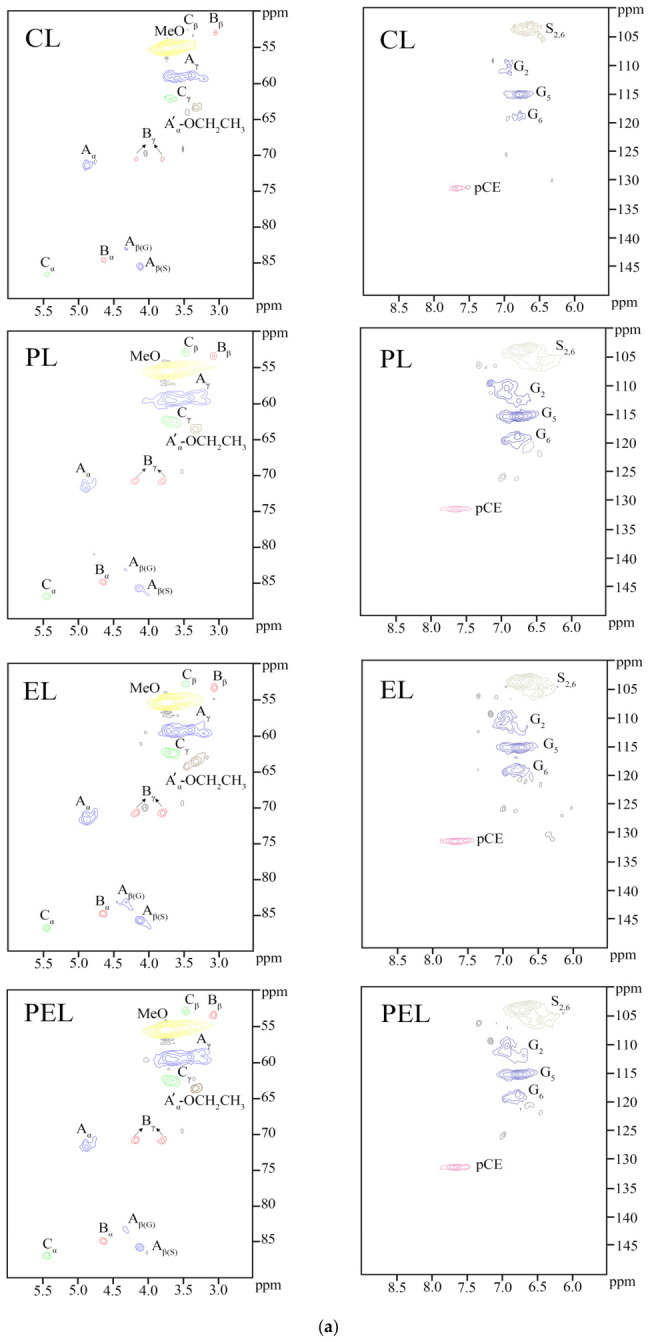

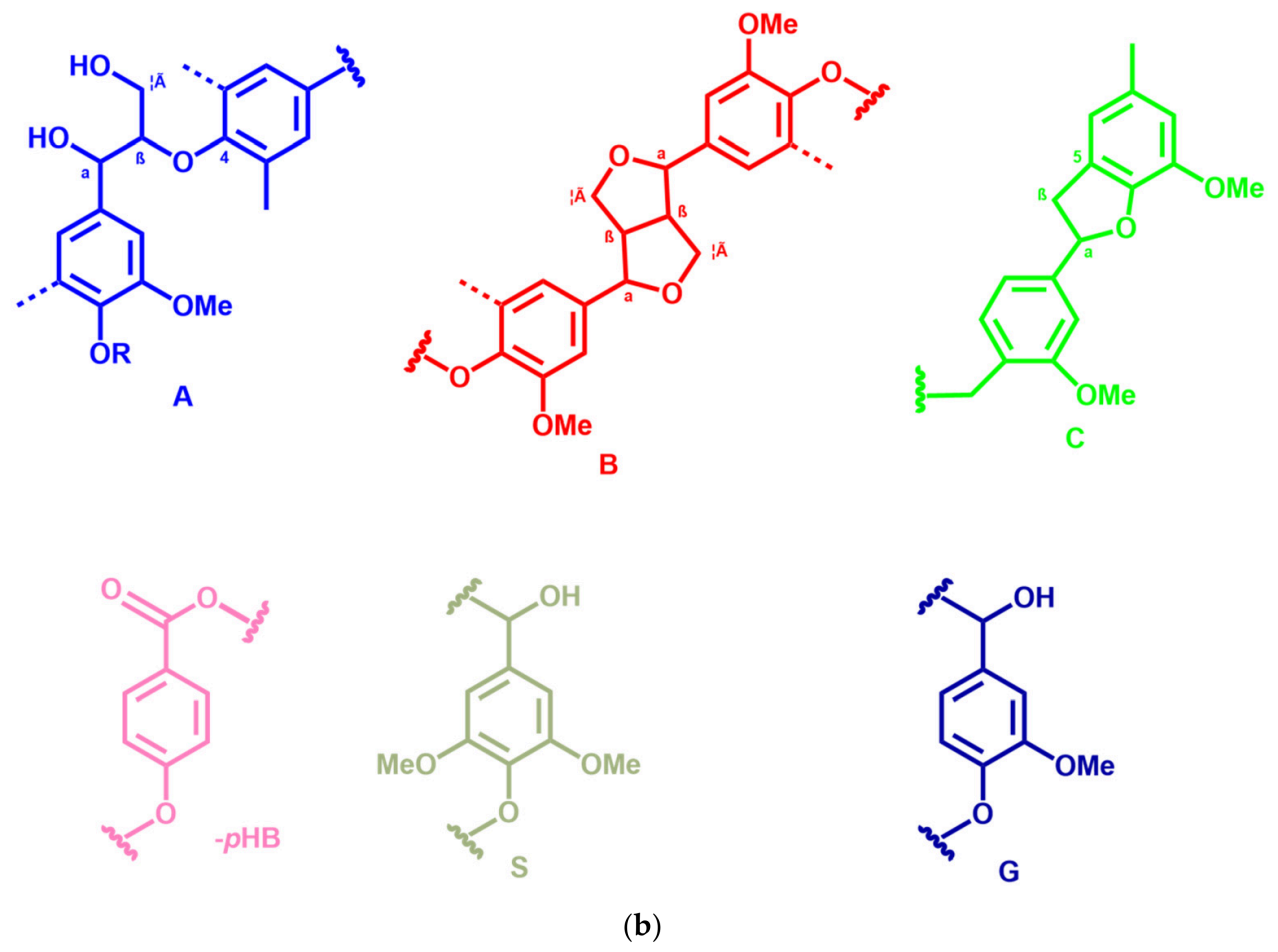
(**a**) 2D-HSQC NMR spectra of the extracted lignin. Lignin extracted from raw material (CL); lignin extracted from hydrothermally pretreated material (PL); lignin extracted from twin-screw extruded material (EL); and lignin extracted from sequentially pretreated material (PEL). (**b**) Typical basic linkage structures and structural units in the side-chain and aromatic regions of the 2D HSQC NMR spectrum of the lignin sample: methoxyl groups (MeO), β-O-4′ aryl ether linkages (A), resinol structures (β-β′, B), phenylcoumaran structures (β-5′, C), C2,6-H2,6 correlations of the p-hydroxybenzoate structure (PB, pHB), syringyl units (S), and guaiacyl units (G).

This mechanism facilitates both the cleavage of ether bonds and the incorporation of the ethoxy group (–OCH_2_CH_3_) into the product side chains, which can be verified by NMR spectroscopy. The γ position of the α-ethoxylated *β*-O-4′ unit was identified in the two-dimensional nuclear magnetic resonance spectrum (63.75/3.32, Figure 7a), confirming the presence of this structural unit. The hydrolysis of the solvent system cleaves the α-aryl ether bond, generating a benzylic carbocation that subsequently reacts with the ethoxy group in ethanol to form an α-ethoxylated *β*-O-4′ unit (A’) [38]. This process can suppress condensation reactions, while the α-ethoxylated site serves as a crosslinking agent for epoxy resins, enhancing the high-value application potential of lignin.

In summary, during organosolv extraction, a portion of water molecules ionize at elevated temperatures to generate H_3_O^+^ and OH^−^. Acetyl groups (–COCH_3_) on hemicellulose side chains are cleaved at elevated temperature, releasing acetic acid (CH_3_COOH), which further ionizes to produce H^+^. Moreover, oxidation of ethanol yields acetaldehyde and acetic acid. The combined presence of these proton sources renders the system mildly acidic (pH ≈ 3.5–4.0). Under these acidic conditions, lignin primarily undergoes ether bond cleavage and condensation reactions among lignin fragments. Ethanol, owing to its strong solvency, preferentially dissolves low-molecular-weight lignin fragments produced by cleavage, facilitating their extraction and thereby mitigating lignin condensation. Additionally, hydrolytic reactions in the solvent system promote cleavage of α-arylether bonds; the resulting benzylic carbocations react with ethoxy groups in ethanol to form α-ethoxylated *β*-O-4′ units, which suppress lignin condensation. Compared with lignin extracted from raw material, lignin obtained via sequential hydrothermal and twin-screw extrusion pretreatment followed by aqueous ethanol organosolv extraction exhibited an increase in phenolic hydroxyl content from 2.10 mmol·g^−1^ to 3.43 mmol·g^−1^, representing a substantial enhancement of reactive hydroxyl groups and favoring the high-value utilization of lignin.

### 3.4. Mass Balance

**Figure 8:**
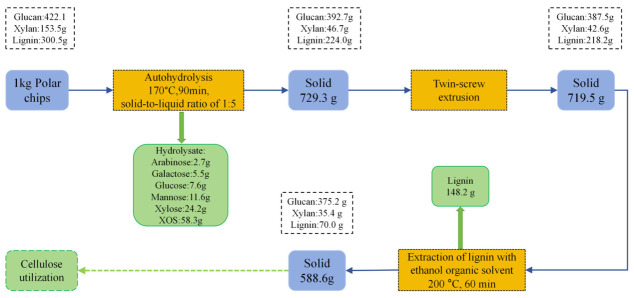
Mass balance of the sequential hydrothermal and twin-screw extrusion pretreatment and organosolv extraction from 1000 g poplar feedstock.

As shown in Figure 8, the mass balance of the sequential hydrothermal and twin-screw extrusion pretreatment followed by organosolv extraction using 1000 g of feedstock was calculated. Following hydrothermal pretreatment, the hydrolysate yielded 24.2 g of xylose and 58.3 g of XOS. A solid residue of 729.3 g was recovered after the hydrothermal process, comprising 392.7 g of glucan, 46.7 g of xylan, and 224.0 g of lignin, indicating substantial xylan removal during the hydrothermal process. The subsequent twin-screw extrusion pretreatment minimally altered the original chemical composition. During the process of the organosolv extraction, 148.2 g of lignin was extracted. This lignin exhibited a relatively high content of phenolic hydroxyl groups, which is favorable for high-value utilization. In total, 24.2 g of xylose, 58.3 g of XOS, and 148.2 g of lignin were obtained from the entire process. Moreover, the remaining solid contained 375.2 g (88.9%) of glucose, which can be further hydrolyzed for sugar production or cellulose-based product manufacturing.

## 4. Discussion

Lignin valorization is constrained by its low phenolic hydroxyl content and structural heterogeneity. Conventional kraft lignin typically contains less than 2.0 mmol·g^−1^ of phenolic hydroxyls, and organosolv lignin generally remains below 2.5 mmol·g^−1^, with extraction yields of approximately 50%. In this study, a synergistic extraction technology was developed, integrating sequential hydrothermal and twin-screw extrusion pretreatment with aqueous ethanol organosolv extraction, which yielded improvements in key metrics. The lignin extraction yield increased to 67.9%, representing a 20.8% improvement over that obtained from direct extraction of the raw material. The total phenolic hydroxyl content increased to 3.43 mmol·g^−1^, corresponding to a 63.3% increase compared to lignin extracted from untreated raw material. Compared with the ethanol-water extraction of European aspen bark (1.60 mmol·g^−1^ phenolic hydroxyl) [47], the phenolic hydroxyl content of the PEL increased by 114% (3.43 mmol·g^−1^). Compared with beech wood organosolv lignin (2.16 mmol·g^−1^) [48], PEL demonstrated a 58.8% increase. It is noteworthy that the pretreatment of poplar with p-toluenesulfonic acid resulted in a phenolic hydroxyl content of 2.24 mmol·g^−1^, accompanied by a 51.4% extraction yield [49]. In a similar study, the combination of hydrothermal and alkali extraction methods led to a phenolic hydroxyl content of 3.09 mmol·g^−1^, with a 51.3% yield [50]. However, the present synergistic approach exhibited a distinct advantage by achieving a higher phenolic hydroxyl content (3.43 mmol·g^−1^) and a higher extraction yield (67.9%), thereby demonstrating a superior process efficiency.

Although the present study demonstrated the effectiveness of the synergistic approach, several limitations should be acknowledged. First, the experimental conditions were established through preliminary simple optimization and reference to existing literature. Subsequent work should focus on developing predictive models driven by in situ characterization (e.g., time-resolved FTIR or NMR monitoring) to elucidate real-time lignin structural evolution. Second, although aqueous ethanol is relatively eco-friendly, the exploration of alternative bio-based solvents such as γ-valerolactone (GVL) and 2-methyltetrahydrofuran (2-MTHF) may further enhance sustainability. Third, upscaling the process requires consideration of continuous extrusion engineering parameters, such as screw configuration and residence time distribution, as well as solvent recovery energy efficiency. Techno-economic assessment (TEA) and life cycle assessment (LCA) are essential for evaluating industrial feasibility. Finally, validation of high-value downstream applications, including carbon fibre precursors, phenolic resins, and functional materials, is imperative for completing the technology value chain.

## 5. Conclusions

This study introduced an innovative and environmentally friendly approach for lignin extraction via sequential hydrothermal and twin-screw extrusion pretreatment followed by aqueous ethanol organosolv extraction. The method significantly enhanced the lignin extraction rate and the phenolic hydroxyl group content, resulting in a 20.8% increase in lignin extraction rate to 67.9% and a phenolic hydroxyl content of 3.43 mmol·g^−1^. This technique addresses the challenges associated with excessive condensation in traditional harsh alkali/high-temperature methods. Hydrothermal pretreatment dissolved hemicellulose and increased porosity under mildly acidic conditions, thereby enhancing accessibility of the cell wall. Subsequent mechanical extrusion further opened the fiber structure and increased specific surface area, thereby improving mass-transfer efficiency. At elevated temperatures, the aqueous ethanol organosolv system promoted acid-catalyzed ether-bond cleavage and ethanolysis, which exposed phenolic hydroxyl groups and suppressed lignin condensation. This method markedly enhanced both the lignin extraction rate and the phenolic hydroxyl content, while simultaneously minimizing cellulose loss, thereby demonstrating high process selectivity. Consequently, it offers an effective strategy for the comprehensive utilization of the three primary components of biomass.

## Figures and Tables

**Table 1:** Pore structure distribution characteristics of raw material and pretreated materials.

Material	Main Peak Aperture (nm)	Main Peak Volume (×10^−3^ m^3^·g^−1^·nm^−1^)	Position of the Secondary Peak (nm)	Feature Summary
C	≈2.0–3.0	≈0.7	4.0–6.0 & 20.0	Micromesoporous pores dominate, multi-level pores are obvious, and the pore size distribution is dispersed
P	≈1.8	≈1.2	8.0	The micropores are concentrated, with the narrowest pore size distribution and the most uniform structure
E	≈2.0	≈1.1	5.0 & 25.0	The double peaks are distinct, and the multi-level pores are prominent
PE	≈1.8	≈1.3	20.0	Medium pores are dominant, while large pores account for a relatively high proportion

Note: Raw material (C); hydrothermally pretreated material (P); twin-screw extruded material (E); sequentially pretreated material (PE).

**Table 2:** Molecular weight and polydispersity of the extracted lignins and milled wood lignins.

Lignin	*M*_w_/(g·mol^−1^)	*M*_n_/(g·mol^−1^)	*M*_w_/*M*_n_
C-MWL	8126	1536	5.29
P-MWL	9266	2946	3.15
CL	3606	1308	2.76
PL	3507	1336	2.63
EL	3265	1139	2.87
PEL	2495	1085	2.30

Note: Milled wood lignin from raw material (C-MWL); milled wood lignin from hydrothermally pretreated material (P-MWL); lignin extracted from raw material (CL); lignin extracted from hydrothermally pretreated material (PL); lignin extracted from twin-screw extruded material (EL); and lignin extracted from sequentially pretreated material (PEL).

**Table 3:** Hydroxyl content in the extracted lignins and milled wood lignins (^31^P-NMR).

Lignin	Aliphatic OH/(mmol·g^−1^)	Syringyl OH /(mmol·g^−1^)	Guaiacyl OH /(mmol·g^−1^)	p-Hydroxyphenyl /(mmol·g^−1^)	COOH /(mmol·g^−1^)	OHphen /(mmol·g^−1^)
C-MWL	2.53	0.49	0.67	0.18	0.14	1.34
P-MWL	2.07	1.13	0.87	0.23	0.16	2.23
CL	2.88	1.01	0.78	0.31	0.14	2.10
EL	2.71	1.05	0.76	0.28	0.13	2.10
PL	2.26	1.80	0.99	0.32	0.20	3.11
PEL	2.41	1.98	1.12	0.33	0.12	3.43

Note: Milled wood lignin from raw material (C-MWL); milled wood lignin from hydrothermally pretreated material (P-MWL); lignin extracted from raw material (CL); lignin extracted from hydrothermally pretreated material (PL); lignin extracted from twin-screw extruded material (EL); and lignin extracted from sequentially pretreated material (PEL).

**Table 4:** Lignin linkage contents in extracted lignins and milled wood lignins (2D-HSQC NMR).

Lignin	*β*-O-4′/%	*β*-*β*′/%	*β*-5′/%	*β*-*β*′/*β*-O-4′	S/G
C-MWL	44.88	6.28	1.54	0.15	1.06
P-MWL	28.59	4.60	4.58	0.24	1.77
CL	23.12	4.58	4.72	0.29	2.81
PL	13.91	3.91	4.57	0.38	1.71
EL	26.10	5.26	4.29	0.27	2.84
PEL	14.38	4.17	4.82	0.39	2.06

Note: Milled wood lignin from raw material (C-MWL); milled wood lignin from hydrothermally pretreated material (P-MWL); lignin extracted from raw material (CL); lignin extracted from hydrothermally pretreated material (PL); lignin extracted from twin-screw extruded material (EL); and lignin extracted from sequentially pretreated material (PEL).

**Table 5:** The extraction yield and hydroxyl content of different lignin extraction methods.

Raw Material	Lignin Extraction Methods	OH_aliph_ (mmol·g^−1^)	OH_phen_ (mmol·g^−1^)	COOH (mmol·g^−1^)	Lignin Extraction Rate/%	Data Source
European aspen *(Populus tremula*) bark	Ethanol/water	4.01	1.60	0.04	--	[47]
European aspen (*Populus tremula*) bark	*n*-butanol/water	3.90	0.83	0.07	--	[47]
Beech wood	Organosolv lignin (OL)	4.26	2.16	0.1	--	[48]
Hybrid poplar	*P*-toluenesulfonic acid treatment	4.12	2.24	0.82	51.4	[49]
Hybrid poplar line NM6 (*Populus nigra* × *Populus maximowiczii*)	Extraction in γ- valerolactone (GVL)	-	-	-	56.5	[50]
Poplar (*Populus tomentosa*)	Enzymatic hydrolysis lignin (EHL)	3.84	0.71	0.11	89.4	[51]
Poplar (*Populus tomentosa*)	Hydrothermal pretreatment combined with alkali extraction	2.25	3.09	0.16	51.3	[51]

## Data Availability

The original contributions presented in this study are included in the article. Further inquiries can be directed to the corresponding authors.

## Abbreviations

The following abbreviations are used in this manuscript:

C	Untreated raw material
P	Hydrothermally pretreated material
E	Twin-screw extruded material
PE	Sequential pretreated material
MWL	Milled Wood Lignin
C-MWL	Milled wood lignin from raw chips
P-MWL	Milled wood lignin from hydrothermally pretreated material
CL	Lignin extracted from raw material
PL	Lignin extracted from hydrothermally pretreated material
EL	Lignin extracted from twin-screw extruded material
PEL	Lignin extracted from sequentially pretreated material
C-EtOH	Solid residue from raw material after lignin extraction
P-EtOH	Solid residue from hydrothermally pretreated material after lignin extraction
E-EtOH	Solid residue from twin-screw extruded material after lignin extraction
PE-EtOH	Solid residue from sequentially pretreated material after lignin extraction
